# Digital screening for postnatal depression: mixed methods proof-of-concept study

**DOI:** 10.1186/s12884-022-04756-2

**Published:** 2022-05-23

**Authors:** Emily Eisner, Shôn Lewis, Charlotte Stockton-Powdrell, Ria Agass, Pauline Whelan, Clare Tower

**Affiliations:** 1 Division of Psychology and Mental Health, University of Manchester, Manchester, M13 9PL UK; 2 Greater Manchester Mental Health NHS Foundation Trust, Manchester, UK; 3 Division of Informatics, Imaging & Data Sciences, University of Manchester, Manchester, UK; 4 St Helens and Knowsley Teaching Hospitals NHS Trust, Rainhill, UK; 5 Manchester University NHS Foundation Trust, Manchester, UK

**Keywords:** Postnatal depression, Digital mental health, mHealth, Smartphone, Maternal mental health, Screening, Mixed methods, Framework analysis

## Abstract

**Background:**

Depression during the postnatal year is prevalent in mothers (17%) and fathers (9%), and suicide is the leading cause of maternal death in this period. Lifelong costs and consequences of untreated postnatal depression (PND) are high due to impacts on infants as well as parents. We aimed to improve access to PND treatment using digital screening. We developed a smartphone app (ClinTouch DAWN-P) that allows parents to monitor their mood daily with the Edinburgh Postnatal Depression Scale (EPDS), uploading responses in real-time to a secure server. We evaluated the app’s feasibility, acceptability, validity and safety in a proof-of-concept study.

**Methods:**

Pregnant women (≥ 36 weeks gestation) and partners were recruited from antenatal services and invited to complete daily EPDS assessments via the ClinTouch DAWN-P app until 6 weeks postpartum. Participants completed standard paper-based EPDS at two time points for validity comparisons. We examined app acceptability and usability at 6 weeks postpartum with qualitative interviews, examined using framework analysis, and the abridged Mobile App Rating Scale (convergent mixed methods design).

**Results:**

Most (96%) eligible pregnant women approached were keen to try the app. Participating mothers (*n* = 15) and partners/fathers (*n* = 8) found the app easy to use, and 91% continued to use it for the full study period. Overall, 67% of daily app-based assessments were completed, with a history of depression predicting lower app usage. Participants suggested modifications to the app and its deployment to improve usability (e.g., extending the response window and including feedback and parenting advice). The validity of app-based responses was confirmed by high agreement with standard EPDS. App-based and paper-based ratings showed perfect agreement in identifying cases of likely PND. There were no serious adverse events relating to app use.

**Conclusions:**

Digital PND screening appears feasible, acceptable, valid and safe. It also benefits from being remotely delivered: we enrolled all participants remotely during the first COVID-19 lockdown. Use of digital screening could address known shortcomings of conventional health visitor-delivered screening such as limited staff time, parental unwillingness to disclose difficulties to a professional, lack of partner/father screening, and language barriers.

**Trial registration:**

The study was prospectively registered (Clinicaltrials.gov: NCT04279093).

**Supplementary Information:**

The online version contains supplementary material available at 10.1186/s12884-022-04756-2.

## Background

Meta-analyses indicate that 17% mothers [1, 2] and 9% fathers [3] experience major depression within the postnatal year. Suicide is the leading cause of maternal death in this period [4]. Although diagnostically identical to depression at other times [5], the consequences [6] and costs [7] of postnatal depression are substantially greater due to adverse effects on infants as well as parents [4, 8–10].

A child’s first two years present a unique window of opportunity to build strong foundations for good future physical and mental health [10]. Parental postnatal depression hugely impacts children’s experiences during those critical days [6]. Children of parents with postnatal depression demonstrate poorer cognitive, emotional and behavioural development, and greater morbidity and mortality [6, 11]. Postnatal depression negatively impacts parent-infant bonding, parental quality of life, health and social functioning [6, 11]. The lifelong consequences of untreated postnatal depression for children and parents cost UK health and social care approximately £1.2 billion per one-year birth cohort [7].

Increasing access to high-quality perinatal mental healthcare is recognised as a key strategic priority for the UK National Health Service (NHS Long Term Plan) [12]. However, in order to offer treatment, services must first identify which parents need it. The Edinburgh Postnatal Depression Scale (EPDS) is a 10-item screening measure which detects postnatal depression with 81% sensitivity and 88% specificity [13]. Current UK guidelines recommend that health professionals consider using two brief screening questions at each postnatal contact and then, if symptoms are detected, use the EPDS. However, national reports [7–9] and our local audit data (unpublished) suggest more than half of cases of postnatal depression currently go undetected and untreated.

A practical, cost-effective solution is needed to increase screening effectiveness, identify postnatal depression in a timely manner and facilitate access to appropriate help. This solution should address known barriers, including:

- Limited staff time for screening, and heterogeneous timing/frequency of pre- and postnatal contacts [14].
- Parental unwillingness to disclose mental health difficulties to health professionals [15].
- Language barriers (e.g. 20% of Manchester adults have a non-English main language) [16, 17].
- Lack of partner/father screening [18]. This is now recommended practice in the NHS Long Term Plan [12] and good practice guidelines [19].

Most (94%) people of childbearing age own a smartphone [20, 21], presenting a clear opportunity for a digital screening solution. We developed the ClinTouch DAWN-P smartphone app, which prompts parents each day to answer the ten EPDS questions. Responses are then uploaded in real time to a secure server where they are accessible to clinicians via a password-protected web interface. Unlike the traditional paper-based EPDS, which asks parents how they have felt over the past week, the ClinTouch DAWN-P app asks parents daily how they have felt over the past 24 h, minimising retrospective recall bias. To our knowledge, this is the first study to test a daily version of the EPDS.

We anticipate that, compared to traditional face-to-face health visitor screening, a smartphone app can make screening more efficient, effective and inclusive, whilst retaining high concurrent validity with traditional screening measures. Specifically:

- More efficient: less resource intensive than staff-delivered screening, saving staff time.
- More effective: more parents are screened, with more regular and frequent assessments.
- More inclusive: an app can be made available in multiple languages and scaled up at relatively low cost, allowing partners/fathers to also be screened. Apps are widely used for everyday purposes, making them a low stigma way of assessing mental health [22].
- High concurrent validity: high agreement between daily app-assessed EPDS and traditional weekly paper-based EPDS responses.

Existing reviews [23–25] and our own systematic search of published studies and clinical trial registries found no studies examining the feasibility and acceptability of repeated-measures smartphone-based postnatal depression screening. For the *antenatal* period, we found two published studies testing one-off, in-clinic depression screening via tablet computer[26, 27] and four studies examining daily [28, 29], weekly [29], monthly [30] or tri-monthly [31] smartphone-based antenatal depression screening [30, 31] or monitoring [28, 29]. We found protocols for studies testing fortnightly email-based [32] or SMS-based [33] mental health and/or substance use screening, but none testing a smartphone app postnatally. We found no studies testing digital screening/monitoring of fathers’/partners’ mental health.

The current mixed-methods proof-of-concept study aimed to examine:

i) The feasibility and safety of using the ClinTouch DAWN-P system to screen for parental depression from late pregnancy (≥ 36 weeks) until 6 weeks postnatally;
ii) App usage patterns, usability and acceptability;
iii) The preliminary validity of daily app-based EPDS assessments, compared to gold standard weekly paper-based reports.

We plan to report additional qualitative data in a follow-up paper exploring participants’ first-person experiences of using an app for postnatal depression screening.

## Methods

### Design

This study had three phases. First, cross-sectional assessments checked eligibility and characterized the sample. Second, participants used the ClinTouch DAWN-P app from ≥ 36 weeks gestation until 6 weeks postpartum. Third, at 6 weeks postpartum, app/study experiences and acceptability were explored using mixed qualitative and quantitative methods. A convergent mixed methods design was appropriate as it enabled us to quantify acceptability across the sample (using a questionnaire) and to explore how to improve the app and study design for future iterations (in qualitative interviews). Quantitative and qualitative findings were integrated narratively using a contiguous approach [34].

Ethical approval was obtained from Greater Manchester East Research Ethics Committee (19/NW/0763) and the study was registered (Clinicaltrials.gov: NCT04279093).

### Participants

Inclusion criteria for pregnant women were: ≥ 36 weeks’ gestation, aged over 18 years, fluent in English, under the care of Manchester University NHS Foundation Trust, informed consent. Exclusion criteria were: current stillbirth, fetal abnormality, or multiple pregnancy. Where present, partners of participating pregnant women were invited to participate. Partner inclusion criteria were: male/female partner of a pregnant participant, aged over 18 years, fluent in English, informed consent.

Pregnant women were recruited from non-emergency antenatal services at St Mary’s Hospital, and via social media adverts. St Mary’s is a large maternity hospital operating across Greater Manchester, an area with high levels of ethnic and cultural diversity (30% identify as a non-White ethnicity [16]) and deprivation (43% of Manchester neighbourhoods are highly deprived [35]).

Senior clinicians (CT, RA) identified potential participants and obtained consent to pass individuals’ contact details to a researcher who completed the informed consent process via phone. Individuals responding to social media adverts contacted the researcher directly. Partners were recruited via pregnant participants. Clinicians and researchers clarified that participation was voluntary. To inform feasibility outcomes, eligible pregnant women or partners who chose not to participate were invited to briefly state their reasons.

### ClinTouch DAWN-P digital screening

#### Digital screening

Android and iOS versions of the ClinTouch DAWN-P smartphone app were available via the app stores during the study. The app alerts the user once per day, using a beep and visual notification, to answer the ten Edinburgh Postnatal Depression Scale (EPDS) items. Each item is displayed in turn along with four possible responses, chosen via radio buttons (supplementary Fig. 1). Items and response options reproduce the questions and sequence of the original EPDS.

App alerts are timed pseudo-randomly once per day between 9am and 7 pm. The user has a 2-h response window in which to complete the EPDS. They can press a ‘snooze’ button to receive a reminder 30 min after the initial alert. Participants rate the items for presence and severity since the previous daily alert. Participants’ responses are wirelessly uploaded in real time to a secure server. The research team can view responses via a password-protected web interface. The app did not meet requirements for registration as a medical device by the Medicines and Healthcare products Regulatory Agency (MHRA).

#### Edinburgh Postnatal Depression Scale

The EPDS is a ten item screening measure, usually used in person by health visitors. The English version has been extensively validated in perinatal women (IPD meta-synthesis: sensitivity 81%, specificity 88% [13]) and validated versions exist in numerous other languages including Urdu [36], Punjabi [36], Arabic [37], Cantonese [38], Persian/Farsi [39], Bangla [40] and Romanian [41]. Seven published studies examining the scale’s validity in partners/fathers report heterogeneous findings [18]. Original and translated EPDS versions ask parents to recall their mood over the past week. The current study is the first to test a daily version of the EPDS.

### Procedure

#### Remote study procedures and participant payment

Participants were enrolled during the first wave of the COVID-19 pandemic, so all study procedures were conducted remotely via a combination of telephone calls, SMS messages, email and post.

Participants received £60 in shopping vouchers for participating: £20 each for baseline and exit phone calls, and a further £20 on completing the app use phase. They also received £10 phone credit per month of the app use phase. Financial incentives were not contingent on a certain number of daily EPDS assessments being completed.

#### Baseline assessment and app training

Following eligibility checks and informed consent, participants answered demographic questions (verbally via phone) about: age, gender, ethnicity, employment status, whether English was their first language, and past psychiatric history. Further information was gathered from pregnant participants’ health records: BMI, past psychiatric history, answers to mental health screening questions at booking appointment, details of current childbirth (mode of delivery, live/still birth, any major obstetric complications), parity (total number of pregnancies reaching viable gestational age).

The researcher then provided verbal and written instructions about how to download, install, configure and use the DAWN-P app. Smartphones were available to borrow if necessary. Participants practiced answering app questions to check they felt confident doing so.

#### App use

Participants were asked to answer daily EPDS questions via the app from study entry (≥ 36 weeks’ gestation) until 6 weeks postpartum. As birth could occur at any point between study entry and 42 weeks’ gestation, the exact length of the app use phase varied from 6–12 weeks’ duration.

To ensure participant safety, app-generated EPDS responses were checked daily by a researcher. Any response of “yes, quite often” or “sometimes” to the EPDS self-harm question (“The thought of harming myself has occurred to me”) was communicated to the participant’s GP within one working day. All participants were informed of this process during consent. Aside from self-harm disclosures (*n* = 1), no other information from app-reported EPDS was communicated to GPs.

Participants received a brief phone call from the researcher after one week of app use, and fortnightly thereafter, to troubleshoot technical difficulties, address queries/concerns and monitor adverse events. Adverse events were recorded, then classified by a consultant obstetrician (CT) as related or unrelated to app use.

Participants completed a conventional paper EPDS after one week of app use and again at 6 weeks postpartum (for validity comparisons with daily app-reported EDPS). If paper EPDS total score was ≥ 12, the researcher sought the participant’s permission to communicate this to their GP. If they declined, information was only communicated if there was risk of harm to self/others. In this proof-of-concept study, information from app-reported EPDS was not sent to health professionals.

#### Exit interviews

In a final phone call at 6 weeks postpartum, app/study experiences and acceptability were explored using a quantitative assessment and qualitative interviews.

##### Quantitative assessment

Participants completed a 12-item version of Mobile App Rating Scale (MARS) [42, 43] prior to the interview. The MARS has high internal consistency (alpha = 0.90) and interrater reliability (ICC = 0.79).

##### Qualitative interviews

All participants, including those who dropped out of the app use phase, were invited to participate in an individual semi-structured interview exploring a) their experiences of the app, b) their experiences of the study, and c) their views on the acceptability of each. Interviews followed a detailed topic guide (available on request) including questions on: app acceptability, look and feel; item content, layout, wording and response format; length and frequency of assessments; worries about app use; how app use fitted with participants’ routines; and other experiences of using the app.

The topic guide allowed flexibility in terms of question order and wording, with probe questions used to prompt further elaboration. The topic guide was updated iteratively. Interviews were conducted by the first author, a female postdoctoral researcher with prior experience conducting and analysing qualitative interviews and with personal, lived experience of postnatal depression. The interviewer had prior phone/email contact with participants throughout the study but never met them in person. Interviews were conducted via phone and were audio recorded and later transcribed verbatim.

### Study outcomes and analysis

#### Overview

The study research questions, outcomes and analyses are summarised in Table 1. Additional details of statistical and qualitative analyses are provided below.

**Table 1:** Proof-of-concept study research questions, outcomes and analysis

Concept	Research question	Outcome(s)	Analysis
Feasibility	Is it feasible to use a smartphone app to screen for parental postnatal depression from late pregnancy until 6 weeks postpartum?	Measures of feasibility: • Study recruitment rate • Reasons for declining to participate • Study dropout rate • Percentage of daily app assessments completed during the app-use phase • Percentage of participants completing ≥ 33% of app assessments • Percentage of participants completing ≥ 50% of app assessments	• Descriptive statistics of all listed measures of feasibility • The a priori “accept” criterion for app engagement was ≥ 33% daily EPDS completed across the sample, with the “target” criterion being 50% of participants submitting 50% of data entries
Patterns of app use	Which parents engage most with the app? How do users’ app use patterns change over time?	• Baseline clinical/demographic information • Percentage daily app use in the app use period • Level of app use per week of the study	• Effects of baseline variables on percentage daily app use examined using Spearman’s correlation (continuous variables), Mann–Whitney or Kruskal–Wallis tests (categorical variables) • Pattern of app completion over time examined using mixed effects models (random effect of participant; fixed effect of time; percentage app completion as the dependent variable)
Validity	Is it valid to use a smartphone app for this purpose?	Data from first and last weeks of app-use phase: • App-generated daily EPDS (up to 7 data-points per person per week, averaged across the week for comparison with paper EPDS) • Gold-standard weekly paper EPDS	Agreement between app and paper EPDS data examined for two separate one-week periods using: • Intra-class correlation (total EPDS scores, continuous variable) • Weighted kappa (individual item scores, ordinal)
Safety	Is it safe to use a smartphone app for this purpose?	• Number of minor and major adverse events during the study	• Descriptive statistics of the number of minor/major adverse events and whether these appear to be are related/unrelated to app use
Usability and acceptability	Is the app easy to use? Do parents find it acceptable to use a smartphone app for this purpose?	• Abridged Mobile App Rating Scale • Qualitative interview data regarding usability and acceptability of the app	• Descriptive statistics of quantitative data from the abridged Mobile App Rating Scale • Framework analysis of qualitative data (a priori themes relating to usability/acceptability)
User experiences (follow-up paper)	What are parents’ experiences of using an app for this purpose? What are parents’ experiences of the study procedures?	• Qualitative interview data regarding experiences of app use and study procedures	• Framework analysis of qualitative data (a posteriori themes), to be published in a follow-up paper

^a^*EPDS* Edinburgh Postnatal Depression Scale

#### Statistical analysis

Statistical analyses were conducted in Stata (version 14.0) and considered statistically significant at *p* < 0.05. As the sample size in this proof-of-concept study was small, no adjustment was made to account for multiple comparisons. The a priori “accept” criterion for app engagement was ≥ 33% daily EPDS completed, and the “target” criterion was 50% of participants completing 50%.

#### Qualitative analysis

The research team conducted a framework analysis of qualitative interviews using a combination of deductive and inductive coding [44]. This was appropriate as we aimed to investigate specific issues regarding app/study acceptability (a priori themes. e.g., app look/feel), and also to explore unanticipated aspects of participants’ experiences (a posteriori themes). A priori themes are reported in the current paper. We plan to report a posteriori themes in a follow-up paper.

The seven stages of framework analysis [44] were followed: transcription, familiarisation, coding, developing a working analytical framework, applying the analytical framework, charting data into the framework matrix, and interpreting the data. Contextual notes written by the researcher at the time of the interview and excerpts from the researcher’s reflective journal informed the analysis.

#### Mixed methods integration

As is common in a convergent mixed methods design [34], we integrated quantitative and qualitative findings narratively using a contiguous approach.

## Results

### Sample characteristics

Table 2 outlines the sample’s demographic and clinical characteristics.

**Table 2:** baseline sociodemographic characteristics

	Baseline variable	Association between baseline variable and percentage app use		
	Frequency (percentage) unless otherwise stated	Test statistic type	Test statistic value	P value
***Self-reported data (all participants, n* = *23)***				
Age (mean, sd)	33.8 (5.2)	ρ	0.50	0.014
Gender				
Female	15 (65.2)	U	42	0.245
Male	8 (34.8)			
Employment^a^				
Full time work	13 (56.5)	Χ^2^	7.50	0.058
Part time work	7 (30.4)			
Home duties	2 (8.7)			
Unemployed	1 (4.4)			
Ethnicity				
Asian or Asian British	2 (8.7)	Χ^2^	3.34	0.362
Black or Black British	2 (8.7)			
Chinese	2 (8.7)			
Mixed race	1 (4.4)			
White British	16 (69.6)			
English language				
Native English speaker	19 (82.6)	U	24	0.256
English as a second language	4 (17.4)			
Owns a smartphone	23 (100.0)	–	–	–
Borrowed a study phone	6 (26.1)	U	48	0.834
Past depression	5 (21.7)	U	18	0.044
Past psychiatric medication	6 (26.1)	U	22	0.042
Past talking therapy	6 (26.1)	U	49	0.889
***Casenote data (for pregnant women with casenotes available, n* = *13)***^b^				
Body Mass Index (mean, sd)	27.1 (9.4)			
Major obstetric complications	2 (15.4)			
Mode of delivery				
Vaginal	6 (46.2)			
Caesarean section	7 (53.9)			
Parity				
One	2 (15.4)			
Two or more	8 (61.5)			
Missing data	3 (23.1)			
Booking appointment: any depression	3 (23.1)			
Booking appointment: any anxiety/worry	5 (38.5)			
Any psychiatric history	6 (46.2)			

^a^Usual employment when not on maternity leave or furloughed

^b^Sample too small for statistical comparison

### Feasibility

**Fig. 1:**
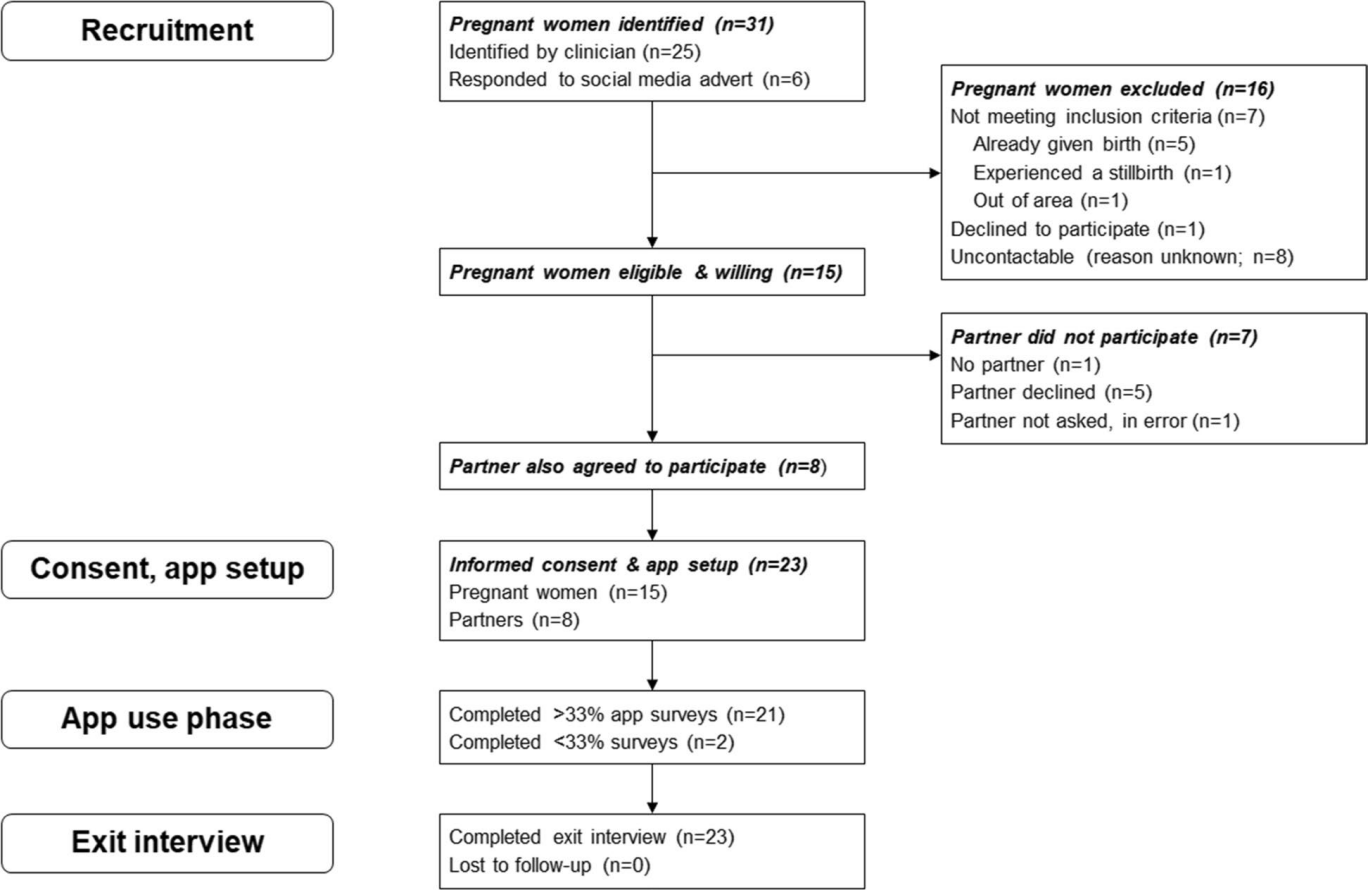
Participant flow through the study

Figure 1 shows participant flow through the study, with details of study recruitment and retention. Of the 24 pregnant women approached by clinicians or identified via social media, 23 (96%) were enthusiastic about participating and one declined without giving a reason. Eight of the 23 could not then be contacted by the researcher. Therefore, the final study sample consisted of 15 mothers (63% of those approached), of whom 8 had partners who were willing to participate (62% of eligible partners). Of the five partners who declined to participate, four gave no reason and one reported they rarely heard their phone due to working with noisy machinery. All participants completed exit interviews and only two participants (9%) dropped out of the app use phase.

**Fig. 2:**
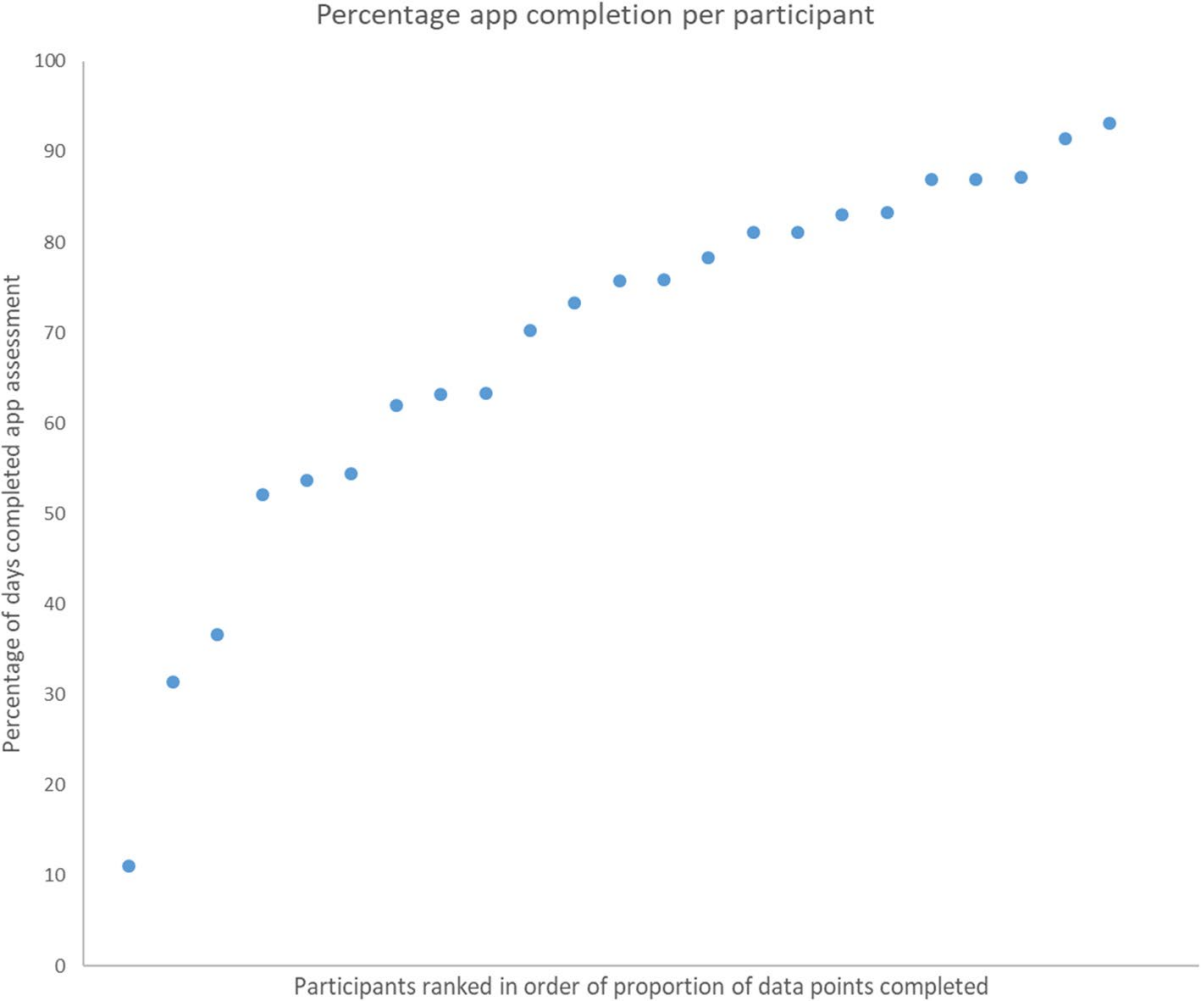
Level of app engagement per participant, averaged across the whole app use period

Figure 2 shows percentage app completion across the sample during the app use period. On average, 67% of daily app-based EPDS assessments were completed. Therefore the study “accept” criterion was met (≥ 33% completed). Most participants (91%; 21/23) completed at least a third of daily app assessments and only slightly fewer (87%; 20/23) completed at least a half of daily app assessments. Therefore the study “target” criterion was met (50% of participants submitting 50% of data entries).

### Patterns of app engagement

Associations between baseline sociodemographic/clinical characteristics and percentage app completion are shown in Table 2. Participant age was significantly correlated with percentage app completion, with older participants completing more daily assessments (albeit with only a moderate effect size). Participants with a self-reported history of depression completed a significantly lower percentage of app assessments (median 54% app completion, range 11%-81%) than those reporting no past depression (median 77.1%, range 31%-93%). Similarly, participants who had previously been prescribed psychiatric medication completed the app significantly less (median 58% app completion, range 11%-81%) than those who did not (median 78%, range 31–93%).

In terms of app engagement over time in the study, participants completed the daily EPDS gradually less as the study progressed (OR = 0.98 per day of follow-up; *p* < 0.001; supplementary Fig. 2).

### Validity

Intra-class correlations between daily app-based and weekly paper-based EPDS total scores were high and statistically significant (Table 3), indicating excellent agreement. Weighted kappa coefficients for individual EPDS item scores ranged from 0.5 and 1.0 (with two outliers of k = 0.25 and k = 0.00). Although all but the outliers were statistically significant, 95% confidence intervals were wide, likely due to the small sample size and low variability in individual item scores. Kappa coefficients for the final week’s EPDS item scores were all higher than those for the same item in the initial week. This may be because there was greater between-participant variability in mood at 6 weeks postpartum (final week) compared to during pregnancy (initial week).

**Table 3:** Comparison of daily app-reported EPDS (mean average of up to 7 days of data) with weekly paper EPDS for the initial week and the final week of the app use period

	Initial week (*n* = 23)		Final week (*n* = 22)	
	ICC^a^/ kappa^b^	95% CI	ICC^a^/ kappa^b^	95% CI
Total EPDS	0.91	0.81 – 0.96	0.97	0.93 – 0.99
EPDS1	0.77	0.41 – 1.00	1.00	1.00 – 1.00
EPDS2	0.66	0.34 – 0.98	0.87	0.59 – 1.00
EPDS3	0.58	0.28 – 0.88	0.70	0.36 – 1.00
EPDS4	0.58	0.27 – 0.89	0.79	0.53 – 1.00
EPDS5	0.82	0.60 – 1.00	0.82	0.62 – 1.00
EPDS6	0.50	0.25 – 0.75	0.87	0.69 – 1.00
EPDS7	0.54	0.18 – 0.90	0.25	-0.22 – 0.72
EPDS8	0.51	0.19 – 0.84	0.63	0.31 – 0.95
EPDS9	0.51	0.17 – 0.85	0.92	0.73 – 1.00
EPDS10	0.00^c^	0.00 – 0.00	1.00^c^	1.00 – 1.00

^a^Two way mixed effects model, absolute agreement; calculated for total EPDS score (continuous)

^b^Weighted Cohen’s kappa, calculated for EPDS individual items (ordinal)

^c^These extreme scores appear to be caused by the vary low variability in participant scores on this item (thoughts of self-harm); only one participant rated this item above zero on the app and/or the paper EPDS

App-based and paper-based EPDS showed perfect agreement in classifying participants as above or below threshold the commonly-used threshold for likely postnatal depression (total EPDS score ≥ 12).

### Safety

During the app use period, one Serious Adverse Event was recorded (hospitalisation for postpartum psychosis), and 4 minor adverse events (1 A&E trip; 3 stayed in maternity hospital for several days (but < 7 days) post birth). None of these adverse events were judged to be related to app use.

### Acceptability and usability

Mean score across items for the abridged MARS was 4.1 (s.d. 0.5) out of a possible score of 5, indicating high overall acceptability/usability. One participant wrote:

*“This app help me understand my mental health. Force me to seek help. I researched a lot about my symptoms…and it’s not a shame to talk about it. I talked to my health visitor, family, GP and finally got medication, which really helped me. I am really grateful to this research and app it help me coming out of sadness, anxiety, depression and negative thinking. Before that I was suffering day and night” [P28F, MARS free-text question]*

**Fig. 3:**
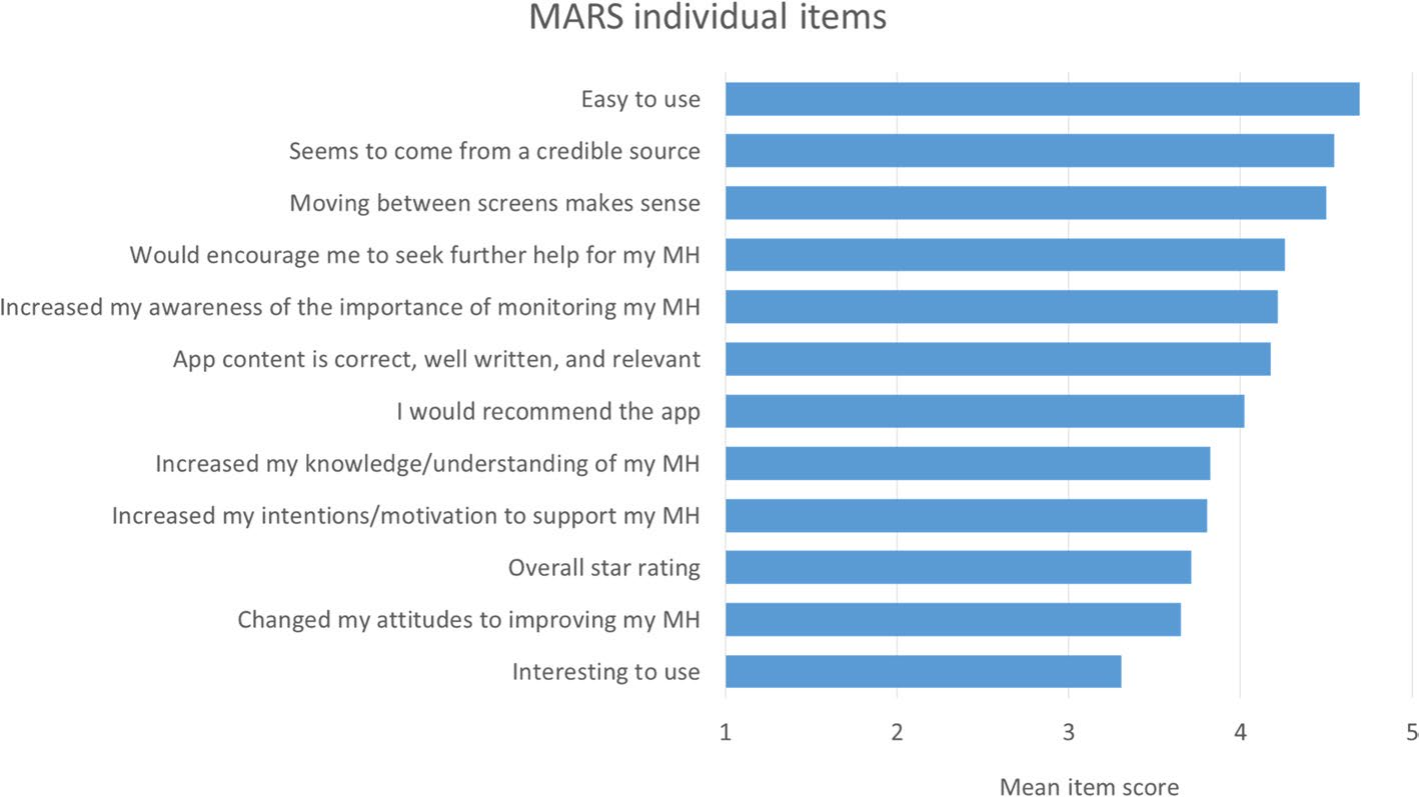
Average responses to abridged MARS items

Responses to individual items (Fig. 3) suggested that, although the app was easy to use (mean 4.7, sd 0.5), the current version was not very interesting to use (mean 3.3, sd 1.1. Qualitative interview data reflected this and included suggestions for making future versions more engaging. A priori themes relating to app acceptability and specifications are described below, with illustrative quotations in Supplementary Table 1.

#### Overall acceptability

Participants considered the core idea of using an app for postnatal depression screening useful and acceptable, although those without symptoms found it less useful, personally. Others were reassured by knowing they would be contacted if their app responses caused concern. Only one person reported finding any aspect of the app worrying – having reported thoughts of self-harm that were passed on to the GP (per protocol), she felt worried about being a burden.

#### App look and feel

Participants enjoyed personalisation options: changing the colour theme and background picture. Most considered the app sufficiently visually appealing, although five said it could be made more modern and colourful.

#### Item content

Generally, participants found item content relevant, appropriate and acceptable. Three suggested expanding content to include questions about functioning and/or other perinatal mental health conditions (e.g. perinatal OCD/psychosis). Several suggested including a free text item allowing users to add details.

#### Item wording and response format

Overall, item wording and response format was acceptable. Most participants considered items easy to answer, although two commented that the wording was more suited to responding weekly (as per original EPDS) than daily. A minority of participants expressed unease about specific EPDS item wording. E.g., three felt that items asking about feeling anxious “for no good reason” minimised their emotional experiences. Several suggested varying the order of questions or response options to reduce repetition. Others suggested varying question wording or rotating through a larger question pool.

#### Response window

Most participants (18/23) said the 2-h response window was not long enough and sometimes prevented them from answering. They would prefer a 4–5 h window or to be allowed to answer any time. Prominent reasons for missing the response window were being busy with child-related tasks, or sleeping while the baby slept. Participants had found it easier to answer within the 2-h response window before their baby’s birth. This emphasises the importance of designing digital tools to meet the needs of specific populations, in this case new parents.

Having missed the limited time window, participants felt frustrated and even guilty, underscoring the need for a longer response window: a short response window is potentially counterproductive (making users feel worse). Participants said they responded less on busy days. They pointed out that, on these busy days, they could probably benefit most from taking time to reflect on their feelings. Similarly, only allowing participants to respond within a limited 2-h period may exaggerate underlying sampling biases (“non-ignorable non-response”) caused by users being less likely to respond on busy days.

#### Alerts and snooze

The app prompted participants at pseudo-random times each day (9am-7pm). Participants commended the decision to avoid notifications during early evening (typically a busy time for parents), although some would have preferred predictable prompts, ideally the same time each day. Eight commented on question frequency: five liked daily questions (aided recall and increased precision) and three found daily questions tedious. As a compromise, several suggested having daily questions initially, later reduced to weekly. Participants found the snooze feature useful but recommended changes its timing. A half an hour snooze was not long enough, and participants requested an extra reminder near the end of the response window.

#### Speed and ease of use

In line with quantitative findings (abridged MARS), all participants reported the questions were quick to complete (30 s to 2 min). Virtually all participants described the app as very easy to use and self-explanatory.

#### Suggested changes to the app

Participants suggested changes to increase app engagement. Several commented that the user is “not getting much back from the app” at present. They were keen for the app to summarise the user’s EPDS responses (e.g. graph or weekly report) and link them to information (e.g. NHS website) and support (e.g. therapist or peer support) based on their responses. Some suggested that adding more general parenting content (e.g. feeding timer) would increase the app’s relevance and interest for parents. Finally, participants suggested ideas to increase the app’s general usability, such as fixing technical glitches, providing a welcome message and back button, and considering gamifying the app.

## Discussion and conclusions

In a busy urban antenatal clinic, most (96%) eligible pregnant women approached were keen to try our newly developed postnatal depression screening app (ClinTouch DAWN-P). Use of the app from up to 4 weeks prenatally to 6 weeks postnatally in 15 mothers and 8 partners showed it to be easy to use and free of adverse effects. Participants were able to suggest modifications to the app and its deployment which would improve usability, such as extending the response window, adjusting question frequency over time, and including feedback and parenting advice. In all, 21 of the 23 participants continued to use the app for the full study period. A mean of 67% of daily app-based assessments were completed, with a history of depression predicting lower app usage. The validity of responses was confirmed by high agreement between app responses and the standard EPDS, both for total scores and individual items. App-based and paper-based ratings showed perfect agreement in identifying cases of likely PND using a standard screening threshold.

In summary, we found this digital solution to be feasible, safe, acceptable and valid. It also benefits from being remotely delivered: we enrolled all participants remotely during the first COVID-19 lockdown. Use of digital screening could address known shortcomings of conventional health visitor-delivered screening such as limited staff time, parental unwillingness to disclose difficulties to a professional, lack of partner/father screening, and language barriers. For example, translated versions of the EPDS can be made available as a menu within the app, broadening access. Operating procedures can be adapted to individual services and clinical pathways: some services use other screening scales (e.g., PHQ-9) and the timing of primary care referral pathways can vary locally. Further work is needed to show how larger-scale adoption of the app translates into longer-term clinical outcomes and to quantify cost savings.

## Supplementary Information

**Additional file 1: Figure S1.** Example Edinburgh Postnatal Depression Scale item, as displayed by the ClinTouch DAWN-P app. **Figure S2.** Patterns of app use over the study duration. **Table S1.** a priori themes describing app acceptability and usability.

## Data Availability

The datasets used and/or analysed during the current study available from the corresponding author on reasonable request.
