# Supplementary material for: Digital screening for postnatal depression: mixed methods proof-of-concept study
Source: BMC Pregnancy Childbirth. 2022 May 23;22:429. doi: 10.1186/s12884-022-04756-2 (PMC9125009; doi:10.1186/s12884-022-04756-2)
Supplement: Supplementary file 1 — Additional file 1: Figure S1. Example Edinburgh Postnatal Depression Scale item, as displayed by the ClinTouch DAWN-P app. Figure S2. Patterns of app use over the study duration. Table S1. a priori themes describing app acceptability and usability. [file 12884_2022_4756_MOESM1_ESM.docx]

**Supplementary material to:**

**Digital screening for postnatal depression: mixed methods proof-of-concept study**

Supplementary Figure 1: Example Edinburgh Postnatal Depression Scale item, as displayed by the ClinTouch DAWN-P app


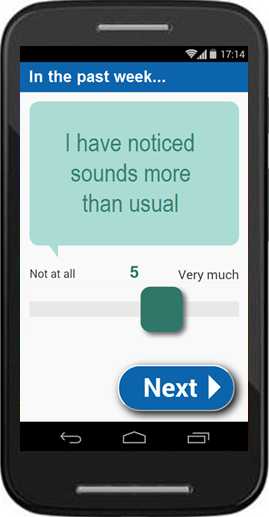

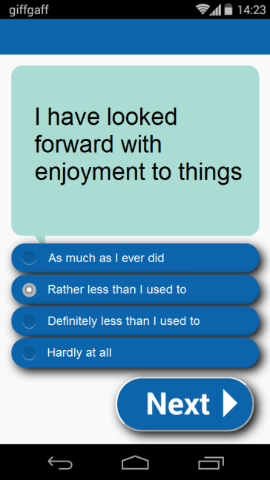


Supplementary Figure 2: Patterns of app use over the study duration


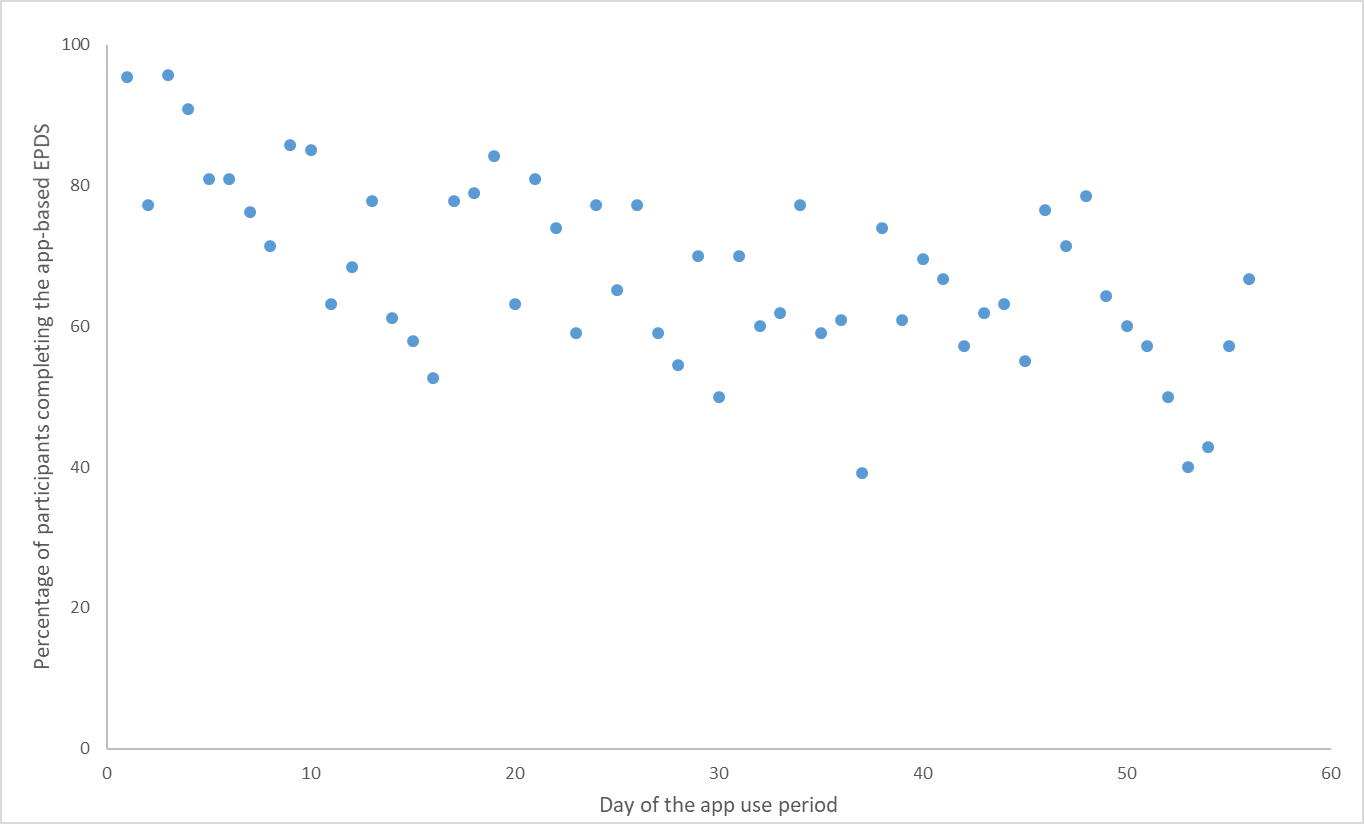


Supplementary Table 1: a priori themes describing app acceptability and usability

| **Theme or sub-theme** | **Quotes [participant number]** |
| --- | --- |
| **Overall acceptability** |  |
| Can see the benefit | “I think the purpose of it’s brilliant. I think it’s definitely a really good foundation to build on” [P02F]  “I thought it was a really really good idea but I did have some technical issues with it… but yeah apart from that I thought it was a really good idea …a really good app.” [P26F]  “I just hope… people get to see it and get to appreciate it anyway because I do like it and I appreciate it so, it’s really helped me these past weeks since I gave birth.” [P09F] |
| Not personally useful as no symptoms | “It was useful when I first signed up to know that I should be aware of my mental health. Um but then after a while yeah it became sort of … well, I feel fine, why am I doing it?” [P12M]  “The questionnaire was just something to complete… I wouldn’t say a chore but…I didn’t *need* it like some people would” [P24F]  “At the moment I’m pretty good really. So… I don’t think I personally would continue to use it” [P27M] |
| Reassuring | “You’ll have it at the back of your mind that you have someone on the app listening to what you have to say… someone that will just reach out to you and ask you what is going on and all that.” [P09F]  “It was quite nice to sort of tick the boxes to say “no actually, I am doing well” and that was quite reassuring in itself” [P07F]  “I guess it maybe kind of reassures you that some … some days are good and some days aren’t as good.” [P27F] |
| Only one participant reported worry about the app | “I was already in hospital by that point but when I’d put down like I’d thought about suicide… I got a text saying it’s gone to your doctor, it was a bit, I don’t know what it was. It just makes you worry, I think. But I obviously knew that would happen… But I just didn’t, I just didn’t like admitting it then.”; “It’s better that someone knows though I spose…I feel a bit ashamed I think at the same time”; “cos I was already in hospital so I didn’t want to, you know, like [laughs slightly] bug anyone else about it. But they were probably already aware anyway so it. I think at the time obviously I felt a bit like a burden” [P12F] |
| **App look, feel and navigation** |  |
| Customisation | “I liked that you could change the colour!” [P02F]  “I’ve changed the background as well so it’s a sunset so it’s like positive yeah” [P08F] |
| Fit for purpose | “Visually it’s very straightforward. It’s very clean looking. There’s not anything extra or confusing on there at all really” [P08F]  “It fits its purpose … Improvement is always good but at the moment I don’t see any issues” [P17M]  “Not anything exciting but not anything boring… I don’t think you need it to be any more than what it is. I think it’s fine as it is” [P11F] |
| Could be more modern/colourful | “It’s functional but not pretty! ... It doesn’t look as polished as you find some other apps look… it just looks a little bit dated I s’pose” [P10M]  “It could do with a little bit more colour, mind. It’s a bit dull.” [P18F] |
| **Item content** |  |
| Overall, item content was relevant, appropriate and acceptable | “The answers were all kind of pinpointing the correct… variations of feelings” [P24F]  “It’s not really that sensitive questions… I think a lot of people won’t mind people asking these questions” [P17M] |
| Could add questions about functioning or other perinatal mental health symptoms | “More in depth questions… like ‘have you looked after yourself today?’…those are the things that you lack doing when you’re going downhill mentally.” [P12F] |
| Could add a free text answer to allow further details to be included | “Some kind of in general free text, or putting things in your own words would be good” [P12M] |
| **Item wording & response format** |  |
| Acceptable, straightforward and easy to answer. | “They were really clear. There was no ambiguity really in the way they were asked.” [P02F] |
| Wording perhaps more suited to weekly than daily response | “They’re like weekly ones really aren’t they and they’re not very specific to a day” [P12F] |
| Certain EPDS items are a little oddly worded | “There have been times during the study where I have been anxious or worried… but it’s not that it’s *not a good reason*.” [P10F]  “Certain questions I felt, for instance…“I’ve been [so] unhappy that I’ve been crying”, they’re kind of a bit extreme.” [P19M] |
| Suggestions for making the questions less repetitive | “I don’t know if it would make any difference to be honest but maybe every week or so change the order of the questions” [P08F]  “Look, the order’s not going to make a difference. But the, what the questions *are*” [P10M]  “Either changing the wording, so it makes you think a bit more, or changing the questions maybe” [P26F] |
| **Response window** |  |
| The 2 hour response window was not long enough | “I kind of just wanted to do it when I was ready” [P02F]  “Having that two hour window was harder... I like to try and leave my phone as far away from me as possible… I had to remember to keep checking it” [P07F] |
| Reasons for missing the response window | “I find it quite easy to miss it because of the timescale it gives you… by the time you’ve fed the baby… and then changed it’s bum…then it might take you nearly two hours to do that! [laughs]” [P11F]  “When the little one arrived…you haven’t got that five minutes so to speak in the two hour window. You just don’t know what, each day’s different” [P24F]  “It’s difficult to remember to go back to them within 2 hours, especially if you’re at work… you can’t be seen to pull your phone out.” [P12M] |
| Frustration at missing the response window | “That was another frustration for me cos it was only a couple of hours…. I easily could be distracted for that whole time and then it would be too late… [smiling] it’d be like “you’ve failed to answer these questions!” and I’d be like “this is making me feel worse!” [laughs]” [P02F]  “I was feeling really bad for the fact that I hadn’t [laughing] answered the questions… it sounds daft but… if things are getting on top of you already then you just don’t really need [laughing] additional stress” [P10F] |
| Less likely to respond on busy days | “I feel like some of the days where you haven’t got the time are the days where you don’t feel as good because everything’s getting on top of you… it would do you some good to maybe just read through those questions again on that day. And reflect a bit” [P11F] |

| **Alerts and snooze** |  |
| --- | --- |
| Alerts could be more predictable | “If you had it at the same time then you’d know… when you were going to expect it.” [P27F] |
| Time of day | “If they were earlier in the morning I thought well I haven’t really had much time to have different feelings [smiling/laughing]… So maybe a little bit later on in the day would be better” [P26F]  “I notice it doesn’t pop up very late in the day which is really good when you’re trying to get everyone to bed and stuff” [P11F] |
| Question frequency | “Daily is really great because you can track a lot more precise[ly]… that person’s emotional condition” [P17M]  “Normally you just do that standard “how are you feeling” thing with the health visitor at eight weeks… I think it’s much easier to consider your mental health on a daily basis…rather than think about the last seven days” [P27F]  “It could be… the beginning six weeks every day and then after that it can drop to maybe once a week… Because the frequency has been decreased I think a lot more people will keep going” [P17M] |
| Snooze feature | “It would be better if… it did it after an hour ‘cause a lot of the time you still are gonna be busy in that half hour if you’ve snoozed it” [P26M]  “If there was a snooze option, with a reason, like ‘at work’ or … I don’t know ‘at the shops’ or something… where you can snooze it and if it says you’re at work, it might prompt again at 6 or you could say like ‘try again in 15 minutes’ …if you’re at the shops.” [P12M] |
| **Speed and ease of use** |  |
| Quick to use | “It takes two minutes to fill out the questionnaire so from that perspective that’s great – the speed and ease of use.” [P10M]  “It’s quite quick, you can go through it quite quickly which is ideal for um a new parent to be honest.” [P19F] |
| Easy to use but boring | “It’s very self-explanatory.” [P11F]  “It was easy to use but maybe a bit too easy… a bit dull!” [P12F]  “It’s clear and you can certainly understand how to use it but it’s very much dry and unengaging.” [P19M] |
| **Suggested changes to the app** |  |
| App does not give much back to the user | “As a user I guess I’m not getting much back from the app.” [P02F]  “There’s no way for [me]… to see how my answers have changed and whether there’s any kind of pattern or curve or anything. So at the moment I just go in, I answer the questions and that’s it” [P10F]  “You’re giving to a database but is someone actually gonna help me if I needed help?” [P26M] |
| Suggested ways for the app to give feedback to the user | **Graph or weekly report**  “What would be really helpful is some kind of visual representation of what you’re putting in. Maybe at the end of the week there’s a graph or some kind of report on overall how the app says you are” [P02F]  “You get the [Fitbit app] weekly report saying how you’ve done this week compared to last week… maybe something like that” [P10F]  **Links to information and advice about mental health**  “It could be better if it had, if on different days it signposted you to different things that could help kind of with your mood er on that day depending on how you answer the questions.” [P19F]  “If you can include what’s the symptoms and what is mental health issues and what triggers it and things like that”; “Suggestions to improve their lifestyle and improve their mental health” [P28F]  **Link to someone to seek help from**  “Either a number they can call or a ‘contact now’ button they can hit and… someone would call them” [P26M]  “Link directly to therapists… ‘Cause at the moment … it gives you an idea of how you’re feeling but it doesn’t take you to what you do if you do feel the negative side of things. You just know that you’re feeling negative, you don’t know what you should do with it [laughs]” [P08M]  “If someone did answer that button of self-harming maybe it could prompt “Do you need to go to A&E? Do you need to call 999? Or 111?”… people don’t think it’s an emergency and it really is” [P12F]  **Peer support**  ““I’m quite a big advocate of peer support over anything else. Um I’ve used it in the past for mental health and it was better than any medication or anything. It’s ‘cause you’re both on the same page… somebody else has been in a bad place and pulled themselves out and they’re then an inspiration… So that sort of community I think is the most beneficial for mental health things in particular. [P12M]  “Maybe a forum button… where you can link in with other users of the app” [P02F] |
| Add more general parenting content | “you know stuff like parents like growth spurts and weaning and stuff like that” [P12F]  “If there were other features in there that were relevant to becoming a new parent it would make it more engaging… If there was something like a timer, a bottle feed timer in there, that you could use to set it for every like four hours or so and then the questions popped up say 20 minutes after one of those timers went off, ‘cause you know the person’s gonna be sat there with a bottle in their hand, not really doing anything else.” [P12M]  “There are these baby banks and things you know where you can get help… it’s like a food bank but for baby stuff. And it just occurred to me: wouldn’t that be useful as part of the other things that this app gives you is maybe just useful stuff to know about. Stuff that’s there to help you.” [P02F]  “Signposting people to resources like ‘Sure start’ or support groups or something for mums, that would, especially for people who are not necessarily, haven’t got necessarily a big support network” [P19F] |
| Suggestions to increase usability | “I think the key thing is though: it just needs to work!” [P10F]  “When you start, say some welcome words… a smiling emoji and things like that. That would be great.” [P17F]  “Sometimes you’ll read it too quickly and click on it. I think what you need is a go back button.” [P19F]  “I do find the… reminders on the badge icons on the apps, I find that a good reminder for me because if I see that there’s a notification. …even if I haven’t seen the notification for whatever reason” [P10F]  “I’m just tryna think of… some way of… erm… like… you know how people like social media, surf sites, do all this, do all that. Erm… whether you could kind of implement that in that way but… for me it was just. It was a bit boring at times…Eh, playing games keeps popping into my head but that’s, that’s no good! But I can't get that out of my head [laughs]” [P19M] |
